# Functional Assessment of Outer and Middle Macular Layers in Multiple Sclerosis

**DOI:** 10.3390/jcm9113766

**Published:** 2020-11-22

**Authors:** Lucia Ziccardi, Lucilla Barbano, Laura Boffa, Maria Albanese, Carolina Gabri Nicoletti, Doriana Landi, Andrzej Grzybowski, Benedetto Falsini, Girolama Alessandra Marfia, Diego Centonze, Vincenzo Parisi

**Affiliations:** 1 IRCCS—Fondazione Bietti, Via Livenza 1, 00198 Rome, Italy; lucia.ziccardi@fondazionebietti.it (L.Z.); vincenzo.parisi@fondazionebietti.it (V.P.); 2 Unit of Neurology, Fondazione Policlinico Tor Vergata, Via Oxford 81, 00133 Rome, Italy; dott.boffalaura@gmail.com (L.B.); maria.albanese@hotmail.it (M.A.); 3 Multiple Sclerosis Clinical and Research Unit, Department of Systems Medicine, Tor Vergata University, Via Montpellier 1, 00133 Rome, Italy; carolgabri@gmail.com (C.G.N.); doriana.landi@gmail.com (D.L.); marfia@uniroma2.it (G.A.M.); centonze@uniroma2.it (D.C.); 4 Department of Ophthalmology, University of Warmia and Mazury, Michała Oczapowskiego 2, 10455 Olsztyn, Poland; ae.grzybowski@gmail.com; 5 Institute for Research in Ophthalmology, Foundation for Ophthalmology Development, Collegium Maius Fredry 10, 61701 Poznań, Poland; 6 Ophthalmology Department, IRCCS—Fondazione Policlinico Universitario A. Gemelli, Catholic University, Largo F. Vito 1, 00168 Rome, Italy; benedetto.falsini@unicatt.it; 7 Unit of Neurology and Neurorehabilitation, IRCCS—Neuromed, Via Atinense 18, 86077 Pozzilli (IS), Italy

**Keywords:** multiple sclerosis, preganglionic retinal elements, photoreceptors, bipolar cells, multifocal electroretinogram, neurodegeneration

## Abstract

The involvement of macular preganglionic elements’ function, during the neurodegenerative process of multiple sclerosis (MS), is controversial. In this case-control observational and retrospective study, we assessed multifocal electroretinogram (mfERG) responses from 41 healthy Controls, 41 relapsing-remitting MS patients without optic neuritis (ON) (MS-noON Group) and 47 MS patients with ON: 27 with full recovery of high-contrast best corrected visual acuity (BCVA) (MS-ON-G Group) and 20 with poor recovery (between 0.2 and 1 LogMAR) of BCVA, (MS-ON-P Group). In the latter Group, Sd-OCT macular volumes and thicknesses of whole and inner and outer retina were measured. MfERG N1 and P1 implicit times (ITs), and N1-P1 response amplitude densities (RADs), were measured from concentric rings (R) with increasing foveal eccentricity: 0–5° (R1), 5–10° (R2), 10–15° (R3), 15–20° (R4), 20–25° (R5), and from retinal sectors (superior, nasal, inferior and temporal) between 0–15° and 0–25°. In the MS-ON-P Group, mean mfERG RADs detected from R1 (0–5°) and from the central nasal sector (0–15°) were significantly reduced (*p* < 0.01) with respect to those of the Control, MS-noON and MS-ON-G Groups. No other significant differences between Groups for any mfERG parameters were found. All Sd-OCT measurements, apart from the inner retina macular volume in the central 1 mm, were significantly reduced in MS-ON-P patients compared to Controls. The functional impairment in the MS-ON-P Group was associated but not correlated with structural changes of the outer and inner retinal layers in corresponding retinal Areas and Sectors. Our results suggest that in MS, exclusively after ON with poor recovery of BCVA, the neurodegenerative process can induce dysfunctional mechanisms involving photoreceptors and bipolar cells of the fovea and of the more central nasal macular area.

## 1. Introduction

Multiple sclerosis (MS) is a neurodegenerative disease, characterized by chronic demyelination of the central nervous system, which can result in visual system involvement including retrobulbar optic neuritis (ON) [1].

The ON event is followed by secondary neurodegenerative processes for retrograde trans-synaptic degeneration [2] that involve retinal ganglion cells (RGCs) and their axons [3] forming the innermost retinal layers (IML). In MS patients, an IML dysfunction has been observed by recording abnormal bioelectrical responses with pattern electroretinogram (P-ERG) [4,5,6] that is a well-known reliable electrophysiological technique for assessing IML function [7].

At the present, it is a debated topic to understand whether the neurodegenerative mechanisms occurring in MS, could involve retinal structures beyond the IML towards the preganglionic elements (i.e., photoceptors, bipolar cells) located in the outer and in middle retinal (O-MR) layers.

The function of preganglionic elements can be assessed by electroretinogram (ERG) recordings [8] that, with its variants, allow us to study the bioelectrical activity of photoreceptor and bipolar cells from the whole retina by Full-field ERG (Ff-ERG) [9], from the central retina by focal ERG (F-ERG) [10] and from multiple localized retinal areas by multifocal ERG (mfERG) [11]. In particular, the mfERG technique provides a topographical map of objective bioelectric responses derived from localized retinal areas, which are driven largely by the cone-related preganglionic components. A “kernel analysis” applied to mfERG responses can be used to assess nonlinear functions of the visual system mainly originating from selected populations of photoreceptors and bipolar cells [12,13,14].

In MS patients, the Ff-ERG cone a- and b- waves’ amplitudes have been found reduced [15,16,17,18], thus reflecting post-phototransduction impairment of the photopic system of the whole retina [16], and, by recording F-ERG, impaired photoreceptoral and post-photoreceptoral responses have been found in the macular area [19].

Regarding the mfERG responses in MS, contrasting data have been reported in the recent literature: in fact, mfERG signals have been found either abnormal [18,20,21] or normal [22], due to different types of MS patients (with or without history of ON), acquisition systems and analysis of recordings and limited sample size.

All this contrasting electrophysiological evidence led us to consider that there are no conclusive findings on whether there is or not an O-MR layers dysfunction or functional expression of the extended neurodegenerative process beyond IML in MS.

Therefore, to add information to the debated topic of preganglionic functional involvement or sparing from neurodegeneration, the aim of our work was to assess the function of preganglionic elements in MS patients with the absence or presence of a history of ON, followed by good or poor recovery of the best corrected visual acuity (BCVA).

We attempted to determine whether an O-MR dysfunction could be detected in the central macular area, or whether it might affect more peripheral retinal regions. In addition, we investigated whether the possible O-MR involvement could be observed in specific sectors (Superior (S), Nasal (N), Inferior (I), Temporal (T)) of the central macular region (0 to 15 degrees) and/or in more eccentric retinal areas within the vascular arcades (0 to 25 degrees).

In addition, a morphological involvement of the outer macular layers in MS patients with history of ON was described [20], but with no clear information whether the morphological changes were related or not to the recovery of BCVA after ON. In order to evaluate the macular morphological changes in MS patients with recovery or not of BCVA after an ON, we recently published a work [23] in which a morphological involvement of the outer macular layers was detectable exclusively in those MS patients with poor recovery of BCVA after ON, whereas when a good recovery after ON was reached, the morphology the outer macular layers was not statistically different from those of Controls. Thus, we believed that, in MS patients with poor recovery of BCVA, it could be interesting to evaluate whether a possible preganglionic macular dysfunction could be associated or not to the above-mentioned morphological changes.

## 2. Materials and Methods

### 2.1. Study Design and Participants

All research procedures described in this work adhered to the tenets of Declaration of Helsinki. The study protocol (CEC/795/14) was approved by the local Ethical Committee (Comitato Etico Centrale IRCCS Lazio, Sezione IFO/Fondazione Bietti, Rome, Italy) and upon recruitment, informed consent after full explanation of the procedure was obtained from each subject enrolled in the study.

Eighty-eight relapsing remitting (RR) MS patients were enrolled at the Visual Neurophysiology and Neurophthalmology Research Unit, IRCCS- Fondazione Bietti referred by the Multiple Sclerosis center of the Tor Vergata University Hospital in Rome, between September 2016 and 20 October 2020.

In order to obtain homogeneous MS Groups (without ON and with ON followed by good or poor recovery of BCVA, see below) the MS patients were selected form a large cohort (*n* = 342) based on the following demographic and clinical characteristics:

1. Age between 28 and 45 years;
2. Diagnosis of RR MS according to validated 2010 McDonald criteria [24];
3. MS disease duration (MS-DD), estimated as the number of years from onset to the most recent assessment of disability, ranging from 5 and 15 years;
4. Expanded Disability Status Scale (EDSS), as ten-point disease severity derived from nine ratings for individual neurological domains [25], ranging from 0 to 3; this score was assessed by two trained [26] neurologists (LaB and MA);
5. Treatment with disease-modifying therapies (DMT) currently approved for preventing MS relapses. DMT considered in our study were Interferon-β-1a, Interferon-β-1b, Peginterferon beta-1a, Glatiramer acetate, Natalizumab, Dimethyl fumarate and Teriflunomide [27];
6. Absence of ON, or a single episode of ON without recurrence, that elapsed from the onset of the disease at least 12 months (ranging from 13 to 20 months) before the inclusion in the study. For MS patients with ON, this criterion was chosen, since it is known that the retrograde degeneration following ON occurs over a period of 6 months [28]. When an MS patient was affected by ON in both eyes, we studied the eye affected longer that met the inclusion criteria;
7. Based on the ophthalmological examination, other inclusion criteria were: mean refractive error (when present) between −3.00 and +3.00 spherical equivalent; intraocular pressure less than 18 mmHg, absence of glaucoma or other diseases involving cornea, lens (lens opacity classification system, LOCS III, stage < 1), uvea, retina; BCVA between 0.0 and 1.0 LogMAR of the Early Treatment of Diabetic Retinopathy (ETDRS) charts; absence of central visual field defects and ability to maintain a stable fixation that allowed performing multifocal ERG (see below); absence of other systemic diseases (i.e., diabetes, systemic hypertension, rheumatologic disorders) that may influence the retinal function.

A Group of selected 41 age-matched healthy subjects (mean age: 40.64 ± 4.83 years, 26 females and 15 males), providing 41 normal eyes, with BCVA of 0.0 LogMAR (mean 0.0 ± 0.0), served as Controls.

The selected MS patients were divided into two Groups for age, MS-DD, EDSS and for previous history of presence or absence of ON.

The 41 MS patients (mean age 41.32 ± 3.72 years, 27 females and 14 males, mean MS-DD 8.53 ± 4.19 years, range 5–20 years; mean EDSS score 1.43 ± 1.06, range 0–3) were without history of unilateral or bilateral clinical signs of ON (i.e., painless reduction of BCVA, contrast sensitivity, color vision and any type of visual field defects) and high-contrast BCVA of 0.0 LogMAR (mean 0.0 ± 0.0). When both eyes met the inclusion criteria, only one eye was randomly chosen for the study. Therefore, we considered 40 eyes from 40 MS patients without ON (MS-noON Group).

The 47 MS patients (mean age 40.64 ± 4.96 years, 29 females and 18 males,) were with previous history of unilateral or bilateral ON (i.e., painless reduction of BCVA -between 0.2 and 1 LogMAR-, contrast sensitivity, color vision and visual field defects). They were further divided in to two Groups on the basis of the recovery of BCVA after ON:

The 27 MS patients (mean age 39.92 ± 4.86 years; 17 females and 10 males; mean MS-DD 9.06 ± 5.58 years, range 5–20 years; mean EDSS score 1.53 ± 1.22, range 0–3) were with previous history of a single unilateral or bilateral ON and with “good” recovery of high-contrast BCVA (0.0 LogMAR; mean 0.0 ± 0.0) after ON. Therefore, we considered 27 eyes from 27 MS patients with ON (MS-ON-G Group);

The 20 MS patients (mean age 41.08 ± 4.66 years; 12 females and 8 males; mean MS-DD 9.96 ± 6.03 years, range 5–20 years; mean EDSS score 1.49 ± 1.18, range 0–3) were with previous history of a single unilateral or bilateral ON with “poor” recovery of high-contrast BCVA (between 0.2 and 1 LogMAR; mean 0.357 ± 0.286) after ON, and reduced P-ERG amplitude with respect to our normative data collected in healthy subjects [29]. Therefore, we considered 20 eyes from 20 MS patients with ON (MS-ON-P Group).

Based on the previous mentioned inclusion criteria, the MS Groups with or without ON were homogeneous for age, MS-DD, EDSS and the MS Groups with ON were homogeneous for number of ON and for the time elapsed from ON. All groups were similar for male/female ratio (see the demographics for each Group in Table 1).

In all MS patients and Controls, the BCVA and the functional condition of the preganglionic elements, located in the 25 retinal degrees by mfERG recordings, were evaluated in the same session during the same day of the examination. In all MS-ON-P patients, a morphological study of the macular layers by Sd-OCT examination was also performed, in addition to mfERG and BCVA evaluations, in the same session during the same day of the examination.

### 2.2. Multifocal Electroretinogram Recordings

The mfERG was recorded by using a modified version of Espion system (Diagnosys UK, LTD; Histon, Cambridge, UK) according to our previously published method [14,30,31] following the 2011 International Society for Clinical Electrophysiology of Vision (ISCEV) standards [11]. Briefly, the multifocal stimulus, consisting of 61 scaled hexagons, was displayed on a high-resolution, black-and-white 32” LCD monitor with a frame rate of 75 Hz. The array of hexagons subtended 50 degrees of visual field (25° radius from the fixation point to edge of display). Each hexagon was independently alternated between black (1 cd/m^2^) and white (200 cd/m^2^) according to a binary m sequence. This resulted in a contrast of 99%. The luminance of the monitor screen and the central fixation cross (used as target) was 100 cd/m^2^. The visual stimulation was performed by correcting BCVA for the distance of the visual stimuli. The m-sequence had 2^13-1^ elements, and total recording time was approximately 8 min. Total recording time was divided into sixteen segments. Between segments, the subject was allowed to rest for a few seconds. Focusing lenses were used when necessary. To maintain a stable fixation, a small red cross target (0.5 degree) was placed in the center of the stimulation field. At every mfERG reported that he/she could clearly perceive the fixation target. The eye’s position was continuously monitored by an in-built video system to track fixation losses.

MfERGs were binocularly recorded in the presence of pupils that were maximally pharmacologically dilated with 1% tropicamide to a diameter of 7–8 mm. Pupil diameter was measured by an observer (LuB) by means of a millimeter ruler and a magnifying lens and stored for each tested eye. The cornea was anaesthetized with Benoxinate eye drops 0.4%. MfERGs were recorded between an active Dawson–Trick–Litzkow (DTL) contact electrode and a reference electrode (Ag/AgCl skin electrode placed on the correspondent outer canthi). A small Ag/AgCl skin ground electrode was placed at the centre of the forehead. Interelectrode resistance was <3 KOhms. After automatic rejection of artefacts and post-acquisition processing done by the in-built Espion software, the first-order kernel response was examined. MfERG responses with a signal to noise ≥3 were accepted for the analysis.

In the analysis of mfERG responses, we considered, for each obtained averaged response, the implicit times (ITs) of the first negative peak (N1) and the first positive peak (P1) measured in milliseconds (msec) and the N1-P1 peak-to-peak response amplitude density (RAD) measured in nanoVolt/degree^2^ (ηV/degree^2^).

**Figure 1:**
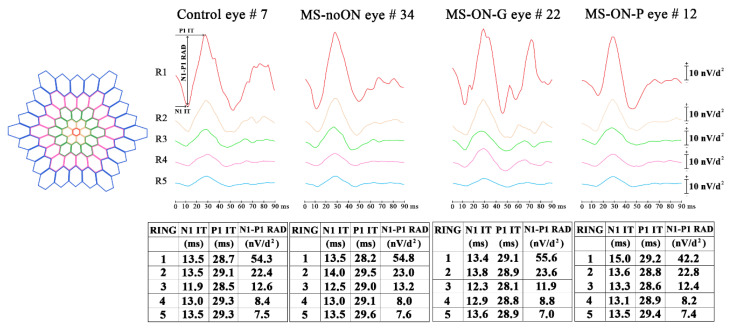
Multifocal electroretinogram averaged recordings obtained in a Control eye (#7), in a patient with multiple sclerosis (MS) without history of optic neuritis (MS-noON#34), and with history of optic neuritis followed by good or poor recovery of visual acuity (MS-ON-G#22 and MS-ON-P#12, respectively) by using ring analysis. For a better comparison, the left eye of representative Control, MS-noON and MS-ON eyes is presented. Ring analysis reports the averaged values of N1 and P1 implicit times (IT, measured in milliseconds -ms-) and of N1-P1 response amplitude density (RAD, measured in nanoVolt/degree^2^ -nV/d^2^-) obtained from five concentric annular retinal regions (rings) centred on the fovea: from 0 to 5 degrees (ring 1, R1), from 5 to 10 degrees (ring 2, R2), from 10 to 15 degrees (ring 3, R3), from 15 to 20 degrees (ring 4, R4) and from 20 to 25 degrees (ring 5, R5).

**Figure 2:**
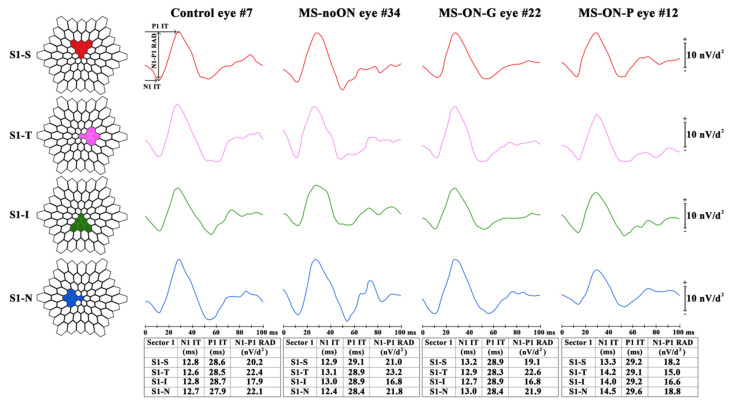
Multifocal electroretinogram averaged recordings obtained in a Control eye (#7), in a patient with multiple sclerosis (MS) without history of optic neuritis (MS-noON#34), and with history of optic neuritis followed by good or poor visual acuity (MS-ON-G#22 and MS-ON-P#12, respectively) by using the sector analysis 1. For a better comparison the left eye of representative Control, MS-noON and MS-ON eyes is presented.

**Figure 3:**
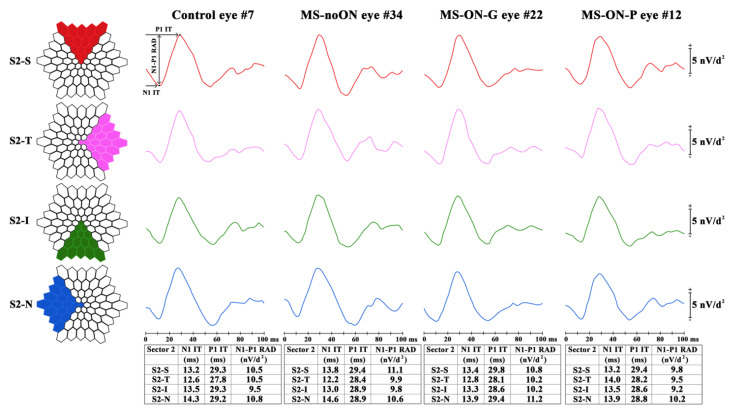
Multifocal electroretinogram averaged recordings obtained in a Control eye (#7), in a patient with multiple sclerosis (MS) without history of optic neuritis (MS-noON#34), and with history of optic neuritis followed by good or poor visual acuity (MS-ON-G#22 and MS-ON-P#12, respectively) by using two different sector analyses 1 and 2. For a better comparison the left eye of representative Control, MS-noON and MS-ON eyes is presented.

We considered three possible retinal topographies to explore the bioelectrical responses derived from specific retinal areas. Data were analyzed as follows:

1. Ring analysis: the averaged response obtained from five concentric annular retinal areas (rings) centered on the fovea: from 0 to 5 degrees (ring 1, R1), from 5 to 10 degrees (ring 2, R2), from 10 to 15 degrees (ring 3, R3), from 15 to 20 degrees (ring 4, R4) and from 20 to 25 degrees (ring 5, R5) (Figure 1).
2. Sector analysis 1: the averaged bioelectrical response obtained from the central macular region up to 15 degrees (0–15 degrees) sectioning it in four sectors: superior (S1-S), nasal (S1-N), inferior (S1-I) and temporal (S1-T) with respect to the fovea. In each sector, we included also the responses obtained from the more central macular area (0–5 degrees) (Figure 2).
3. Sector analysis 2: the averaged bioelectrical response obtained from the retinal area from the fovea up to 25 degrees (0–25 degrees) sectioning it in four sectors: S2-S, S2-N, S2-I and S2-T with respect to the fovea. In each sector, we included also the responses obtained from the more central macular area (0–5 degrees) (Figure 3).

### 2.3. Sd-OCT Assessment

In all MS-ON-P patients, the macular morphology was evaluated by the RTVue-100 Sd-OCT device, following our recently published method [23].

Segmentation analysis was performed in order to measure the macular volume (MV) and macular thickness (MT) of whole, inner and outer retinal layers (WR, IR and OR, respectively) from concentric areas corresponding to the ETDRS topographical map:

(1) the 1 mm central area (named as Area 1, directly provided by the Sd-OCT machine)
(2) the middle 1–3 mm ring (named as Area 2, obtained by subtracting from the displayed volume within 3 mm the ones within the 1 mm),
(3) the external 3–6 mm ring (named as Area 3, obtained by subtracting from the displayed volume within 6 mm the one within 3 mm directly provided by the Sd-OCT machine),
(4) the whole 6 mm area (named as Area 1 + Area 2 + Area 3, directly provided by the Sd-OCT machine).

We also performed a sectorial segmentation analysis of the S, T, I and N sectors within 6 mm (averaging the three values of MV and MT displayed on the machine within the 0.5, 1 and 3 mm of radius from the fovea).

This allowed to compare the electrophysiological data to the morphological ones from corresponding localized retinal areas [32].

Therefore, we considered WR, IR and OR MVs and MTs measured in Area 1 corresponding to mfERG R1, in Area 2 corresponding to mfERG R2, in Area 3 corresponding to mfERG R3 and in Area 1 + Area 2 + Area 3, corresponding to mfERG R1 + R2 + R3. Accordingly, we also compared WR, IR and OR MV and MT values from S, T, I, N sectors to the corresponding mfERG data from Sector analysis 1 (see above).

### 2.4. Statistical Analysis

We assumed a Gaussian distribution of our data. The normal distribution was assessed by using the Kolmogorov-Smirnov test.

The differences of age, MS-DD, EDSS between MS-noON, MS-ON-G and MS-ON-P Groups were evaluated by the one-way analysis of variance (ANOVA). The differences of the number of ON and the time elapsed from the ON between MS-ON-G and MS-ON-P Groups were evaluated by the ANOVA.

Considering each different mfERG retinal topography (Ring, Sectors 1 and Sectors 2 analyses), the differences of mfERG N1 and P1 IT and N1-P1 RAD mean values between Controls, MS-noON, MS-ON-G and MS-ON-P Groups were evaluated by ANOVA. In addition, mean values of segmented MV and MT from all Areas and Sectors within 6 mm detected in MS-ON-P Group were compared to those of Controls by ANOVA. In MS-ON-P Group, Pearson’s test was used to linearly correlate the values of BCVA with those of mfERG parameters and to correlate the individual mfERG values with the segmented MV and MT ones from corresponding retinal Areas and Sectors.

Since for each considered mfERG and OCT parameter, a multiple comparison between Groups (6 comparisons: Control vs. MS-noON Groups, Control vs. MS-ON-G Groups, Control vs. MS-ON-P Groups, MS-noON vs. MS-ON-G Groups, MS-noON vs. MS-ON-P Groups and MS-ON-G vs. MS-ON-P Groups) was performed, the value of statistically significance was calculated by: *p* = 0.05/number of comparison: 0.05/6 = 0.0082. Therefore, we rounded up to a p-value lower than 0.01 to be considered as statistically significant. Minitab 17 (version 1) software was used for statistical analysis.

## 3. Results

### 3.1. Demographic and Clinical Features

In Table 1 are reported the demographic and clinical features observed in Controls, MS-noON, MS-ON-G and MS-ON-P Groups. The descriptive statistics of age, MS-DD and EDSS values were not significantly different between MS-noON, MS-ON-G and MS-ON-P Groups. The descriptive statistics of number of ON and the time elapsed from the ON were not significantly different between MS-ON-G and MS-ON-P Groups.

### 3.2. Multifocal Electroretinogram Ring Analysis

Examples of averaged mfERG recordings from five rings (R1, R2, R3, R4 and R5), obtained in representative Control (#7), MS-noON (#34), MS-ON-G (#22) and MS-ON-P (#12) eyes, are presented in Figure 1.

In Table 2 are reported the mean values of N1 and P1 IT and of N1-P1 RAD detected in the five rings (R1, R2, R3, R4 and R5) in Control, MS-noON, MS-ON-G and MS-ON-P Groups and the relative statistical analysis between Groups.

On average, when we considered the mean values of N1 and P1 IT obtained in the central retinal areas (R1, R2 and R3, 0 to 15 degrees) and in the more peripheral retinal areas (R4 and R5, 15 to 25 degrees), not statistically significant (*p* > 0.01) differences between all Groups were found.

The mean values of N1-P1 RAD obtained in the most central retinal areas (R1, 0–5 degrees) in MS-noON Group were not statistically (*p* > 0.01) different with respect to those of Controls. In MS-ON-G Group, the mean values of N1-P1 RAD were not significantly (*p* > 0.01) different when compared to those of Control and MS-noON Groups; by contrast, in MS-ON-P Group, the mean values of N1-P1 RAD were significantly (*p* < 0.01) reduced with respect to the ones from Control, MS-noON and MS-ON-G Groups; the reduction of the individual N1-P1 RADs were not significantly correlated (*p* > 0.01) with the corresponding values of BCVA.

In MS-noON, MS-ON-G and MS-ON-P Groups, the mean values of N1-P1 RAD obtained in the other areas (R2, R3, R4 and R5) were not statistically (*p* > 0.01) different with respect to those of Controls, and not statistically significant (*p* > 0.01) differences were found between MS Groups.

### 3.3. Multifocal Electroretinogram Sector Analysis 1 (0–15 Degrees)

Examples of averaged mfERG recordings from four sectors superior (S1-S), temporal (S1-T), inferior (S1-I) and nasal (S1-N) within 15 degrees of foveal eccentricity, obtained in representative Control (#7), MS-noON (#34), MS-ON-G (#22) and MS-ON-P (#12) eyes, are presented in Figure 2.

Sector analysis 1 reports the averaged values of N1 and P1 IT and of N1-P1 RAD obtained from four macular areas enclosed between 0 and 15 degrees with respect to the fovea on the basis of the retinal topography: superior (S1-S), temporal (S1-T), inferior (S1-I), nasal (S1-N). The bioelectrical responses obtained from the central 0–5 degrees were enclosed in the sector analysis 1.

In Table 3 are reported the mean values of N1 and P1 IT and of N1-P1 RAD detected in the four central sectors (S1-S, S1-T, S1-I, S1-N) in Control, MS-noON, MS-ON-G and MS-ON-P Groups and the relative statistical analysis between Groups.

On average, when we considered the mean values of N1 and P1 IT obtained in the central sectors (S1-S, S1-N, S1-I, S1-T) not statistically significant (*p* > 0.01) differences between all Groups were found.

The mean values of N1-P1 RAD obtained in these sectors in MS-noON Group were not statistically (*p* > 0.01) different with respect to those of Controls.

In MS-ON-G Group, the mean values of N1-P1 RAD from all four sectors were not significant (*p* > 0.01) different when compared to those of Control and MS-noON Groups. By contrast, in MS-ON-P Group, while mean values of N1-P1 RAD detected in S1-I, S1-T and S1-S were not significantly (*p* > 0.01) reduced with respect to Control, MS-noON and MS-ON-G ones, a significant (*p* < 0.01) reduction of N1-P1 RADs in the S1-N sector was observed as compared to Controls, MS-noON and MS-ON-G Groups.

The individual reduced N1-P1 RAD values from S1-N sector in MS-ON-P eyes were not significantly correlated (*p* > 0.01) with the corresponding values of BCVA.

### 3.4. Multifocal Electroretinogram Sector Analysis 2 (0–25 Degrees)

Examples of averaged mfERG recordings from 4 sectors (S2-S, S2-T, S2-I, S2-N) within 25 degrees of foveal eccentricity in representative Control (#7), MS-noON (#34), MS-ON-G (#22) and MS-ON-P (#12) eyes are presented in Figure 3.

Sector analysis 2 reports the averaged values of N1 and P1 IT and of N1-P1 RAD obtained from four retinal areas from 0 to 25 degrees based on the retinal topography: superior (S2-S), temporal (S2-T), inferior (S2-I), nasal (S2-N), with respect to the fovea. The bioelectrical responses obtained from the central 0–5 degrees were enclosed in the sector analysis 2.

The mean values of N1 and P1 IT and of N1-P1 RAD detected in the 4 sectors (S2-S, S2-T, S2-I, S2-N) in Control, MS-ON and MS-noON Groups and the relative statistical analysis between Groups are reported in Table 4.

On average, the mean values of N1 and P1 IT and of N1-P1 RAD detected in all sectors (S2-S, S2-T, S2-I, S2-N) in MS-noON, MS-ON-G and MS-ON-P Groups were not statistically (*p* > 0.01) different when compared with those of Controls. In MS-ON-G and MS-ON-P Groups the mean values of N1-P1 RAD from all four sectors were not significantly (*p* > 0.01) different when compared to those of Control and MS-noON Groups. Furthermore, not statistically significant differences (*p* > 0.01) were found when mean N1-P1 RADs were compared between MS-ON-G and MS-ON-P Groups in all sectors.

### 3.5. Morphological Data in MS-ON-P Group and Correlations with mfERG Findings

**Figure 4:**
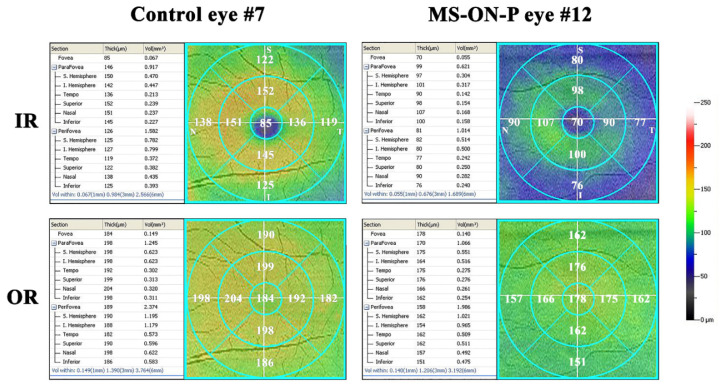
Examples of Early Treatment of Diabetic Retinopathy (ETDRS) topographical map of macular volume and thickness values of inner (top) and outer (bottom) macular layers (IR and OR, respectively) obtained in left eyes of a representative Control (#7) and of a multiple sclerosis patient with history of optic neuritis followed poor recovery of visual acuity (MS-ON-P#12) by using Sd-OCT. On the left side of the ETDRS maps, the volume and thickness numerical values for each sector are reported. On the right side of the Figure, a colorimetric scale is provided to display the macular thickness values. The macular volume and thickness values were measured from concentric circular Areas (Area 1: the 1 mm central area; Area 2: the middle 1–3 mm ring; Area 3: the external 3–6 mm ring; Area 1 + 2 + 3: area within 6 mm) and Sectors (Superior, S; Temporal, T; Inferior, I; Nasal, N) within 6 mm (averaging the three values of macular volume (MV) and macular thickness (MT) displayed on the machine within the 0.5 mm, 1 mm and 3 mm of radius from the fovea). With respect to Control eye, MS-ON-P eye shows reduced MV and MT values in both inner and outer retinal layers (IR and OR) macular layers in each Area or Sector.

Examples of Sd-OCT map of MV and MT of OR and IR macular layers evaluated in representative Control (#7) and MS-ON-P (#12) eyes are presented in Figure 4.

In Table 5 are reported the mean values of segmented Sd-OCT MV and MT of WR, IR and OR measured in Area 1, Area 2, Area 3 and Areas 1 + 2 + 3 in Controls and in MS-ON-P patients. We found a statistically significant difference (*p* < 0.01) between these Groups for all structural values but the IR MV from Area 1.

In Table 6 are reported the mean Sd-OCT MVs and MTs of WR, IR and OR measured in the S, T, I and N Sectors within 6 mm from the fovea in Controls and in MS-ON-P patients. We found a statistically significant (*p* < 0.01) reduction of all morphological parameters from each Sector in MS-ON-P with respect to Controls.

In MS-ON-P patients, we found not significant (*p* > 0.01) linear correlations between the reduced mfERG RADs from Ring 1 with the reduction of OR MV and MT from Area 1. No other significant correlations between mfERG parameters from other Rings with Sd-OCT values from corresponding retinal areas were found.

When we linearly correlated the mfERG data from Sectors-S1 with the corresponding MV and MT individual values from S, T, I, N Sectors, we found not significant linear correlations between the S1 ITs and RADs and WR, IR and OR MVs and MTs. The results of these statistical linear correlations are reported in Table 7.

## 4. Discussion

The purpose of this study was to assess the function of preganglionic elements in MS patients, without and with history of ON, adding information on the debated topic of potential O-MR layers dysfunction, expression of the extension or sparing from neurodegenerative process beyond IML in MS.

We studied by mfERG the function of O-MR elements located in different areas of the central macula (0 to 15 degrees) or more peripheral retina within the arcades (0 to 25 degrees), topographically distinguished in rings or sectors. Our results apply to MS Groups with or without ON highly homogeneous for age, MS-DD, EDSS, and when present for number of ON and for the time elapsed from ON to the BCVA and mfERG assessment, differently from all previous reported studies in the literature [18,20,21,22].

In addition, since a morphological impairment of macular OR has been described in MS-ON patients [20] and in our recent work was confirmed to be detectable exclusively in those MS-ON patients with poor recovery of BCVA [23], we also evaluated in MS-ON-P patients whether a possible preganglionic macular dysfunction could be associated or not to structural OR changes for corresponding retinal areas.

Our mfERG findings showed not statistically significant differences of N1 and P1 IT values in all Groups (MS-noON, MS-ON-G and MS-ON-P) in any considered central circular areas (R1, R2, R3) or sectors (S1-S, S1-T, S1-I, S1-N) and more peripheral circular areas (R4, R5) or sectors (S2-S, S2-T, S2-I, S2-N) either when responses were compared to Controls or with MS Groups. As for N1-P1 RAD values, we found statistically significant (*p* < 0.01) differences in MS-ON-P Group compared to Controls, MS-noON and MS-ON-G only when analyzing responses from Ring 1 (0–5 degrees) and from the S1-N sector, which covers the 0–15 central degrees area. In all other examined central or peripheral rings or sectors, we did not find any significant difference in the values of N1-P1 RAD between Groups. Our results indicate that photoreceptors and bipolar cells of the central fovea, as well as of the more central nasal macular sector within 15 degrees, are functionally impaired in MS only in occurrence of ON and when full recovery of BCVA is not achieved. These results do not apply either to MS-noON nor MS-ON-G Groups, thus confirming that the preganglionic element dysfunction is independent from the event of ON in itself.

As mentioned above, contrasting data are reported in literature about the potential functional involvement of O-MR layers in the MS degenerative process, depending on MS classification, presence or absence of ON and different mfERG signal analyses. As stated by Hanson et al. [33] “similarities or differences between findings in the central and peripheral retina are yet to be definitively elucidated in MS”, and therefore we thought reasonable to study O-MR function in our patients by applying not only the standard ring analysis, but also the more innovative sector analyses previously used in other neurodegenerative diseases [30,34].

In MS-noON Group, we found a functional integrity of O-MR elements, in agreement with the results of a previous mfERG study [22] in which, by using the ring analysis, normal function of preganglionic elements in eyes without ON and normal high-contrast visual acuity was found. By contrast, our results differ from those by Saidha et al. [20], who found in five MS-noON patients with an abnormal OCT macular thickness and normal visual acuity, normal mfERG latencies with reduced P1 amplitude. As for the comparison of sector analysis results in MS-noON, Boquete et al. [35] studied, by using a more refined mfERG analysis method, a small cohort of newly diagnosed MS patients with less than 6 months from their first symptoms and no ON. They found an impairment of O-MR function exclusively in the supero-temporal quadrant of the macula. In our study, we analyzed the mfERG responses sectioning the central macular region up to 15 degrees (0–15 degrees, sector analysis 1) and the whole macular area up to 25 degrees (0–25 degrees, sector analysis 2) in four sectors (superior, temporal, inferior and nasal). By adopting this different way to analyze mfERG sector responses [30,34], we did not find statistically significant differences between Controls and MS-noON. Because the exact protocol used by Boquete e al. [35] could not be replicated in our study since, as stated by the Authors [35,36], this method is currently only for research purposes and it is not a commercially available equipment; we could not confirm their data in MS-noON eyes. As for the “primary retinal pathology” process in MS-noON eyes [20], recalled also by Fairless et al. [37], the presence of neuro-retinitis phenomena could interfere with the results. This point therefore needs to be confirmed by a large study cohort.

In a similar cohort of MS-noON patients, we [23] recently observed an absence of WR and IR MVs reduction, and, differently from Saidha et al. [20], we detected that OR MV and MT values were not significantly different from Controls. Taking in account this evidence, our mfERG results may indicate that in MS-noON patients an absence of outer macular layers’ morphological involvement together with an absence of O-MR dysfunction can be hypothesized.

In the MS-ON-G Group, when measuring mfERG RADs, we also found absence of O-MR dysfunction either by rings or sectors analyses. Our findings diverge from Hanson et al. [18] who evidenced slight abnormal mfERG responses suggesting inhibitory bipolar cell dysfunction in a mixed cohort of clinically isolated syndrome, primary progressive MS and RR MS eyes, with some cases of ON, and recovery of BCVA. In a very recent study, Filgueiras et al. [21] suggested OR dysfunction based on the exclusive findings of significant shorter mfERG N1 and P1 implicit times in MS with and without ON, and concluding that mfERG may help in differentiating MS-ON from “neuro-myelitis optica” spectrum disorder. In agreement with the commentary by Hanson et al. [33], we also considered as questionable the finding by Filgueiras et al. [21], since “anticipated” N1 and P1 latencies that were on average 1 msec shorter than Controls, cannot be considered as electrophysiological evidence of supernormal bipolar function in MS patients. In addition, in their work, the decision of not including in the mfERG analysis the R5 areas could have affected latency results. Finally, the Authors [21] did not correct their p-values for multiple testing, considering the high number of statistical comparisons, thus overestimating the significance of their results.

As for the MS-noON Group, in a similar cohort of MS-ON-G patients, we recently observed significantly reduced WR and IR MVs and MTs, with OR values similar to Controls; an extensive explanation of these findings was previously discussed [23]. Hence, the presence of mfERG values similar to Controls in this Group, and also considering the previously observed [23] absence of OR morphological impairment, led us to believe that in MS patients with previous history of ON and good recovery of BCVA there are structural changes involving IR but not OR, with also normal O-MR function.

In MS-ON-P Group, together with the above-mentioned mfERG changes (reduced R1 and S1-N RADs), we found, in agreement with our previous work [23], a significant reduction of WR, IR and OR MVs and MTs as compared to Controls. The interpretation of these morphological findings was given elsewhere [23].

These observed reduced R1 RAD values let us consider that when BCVA recovery after ON is poor, the wiring of retinal circuitry in the fovea, where the cones and the RGCs have the highest density [36,38], can be severely impaired. This foveal dysfunction was not significantly correlated with the reduction of OR MV and MT values in the central Area 1 and this might suggest that the O-MR dysfunction is associated but not linearly correlated to the OR morphological involvement. In addition, since not significant correlations between the reduced R1 RADs and the reduction of IR MV and MT values in the central Area 1 were found, it could be hypothesized that the morphological involvement of the inner macular layers does not influence the function of the O-MR layers.

All these findings could have different explanations. First, the absence of a perfect anatomical overlapping between the stratified measurements by mfERG and Sd-OCT assessments. For instance, when segmenting IR and OR layers, our RTVue-100 device software automatically divides the inner and outer neurosensory retinas at the boundary between the inner nuclear layer (INL) and the outer plexiform layer (OPL). The OR encloses the OPL, the outer nuclear layer, and the photoreceptor layer. The IR examines the retinal nerve fiber layer (RNFL), the ganglion cells/inner plexiform layer (GC/IPL), and the INL. On the other hand, the mfERG system allows us to record the bioelectrical activity driven mainly by cones and bipolar cells, specifically mfERG response amplitude values are more correlated with photoreceptors activity whereas peak timing is more associated with the contribution to the signal by bipolar cells [39]. Thus, it could be that as the nuclei of the bipolar cells located into the INL (enclosed in our IR segmentation analysis and resulted reduced) and the relative bioelectrical activity is mainly represented by the mfERG ITs (resulted similar to Controls), there is a not perfect colocalization between the structural and functional tests of the same elements. This could explain the absence of correlation between reduced RADs and reduced OR MV and MT values, as well as the absence of correlation between normal P1 ITs and reduced IR MV and MT values in MS-ON-P patients. A second explanation could be related to the sample of MS-ON-P patients enrolled in the present study. We enrolled a high homogeneous number of 20 patients with MS-ON and poor recovery of visual acuity. Eventually, results from a larger cohort of patients may give different results and different correlations between morpho-functional parameters.

By contrast, since in the more peripheral areas (Area 2 and Area 3) we detected normal mfERG responses (ITs and RADs), but reduced WR, IR and OR MVs and MTs, it should be hypothesized that this morphological involvement is not sufficient to induce functional changes, as suggested by the lack of correlation between mfERG and Sd-OCT data, as reported in Table 7.

In these patients, the observed macular functional changes were not significantly related with the reduced BCVA, as well as we recently reported [23] that the reduced recovery of BCVA is also independent from the morphological condition of the outer macular layers but is correlated with the morphological impairment of the inner macular layers.

Our findings of abnormal mfERG responses specifically in the S1 nasal sector links with Boquete et al. [35] findings (reduced first order kernel RADs in the temporal sectors for their right eyes). The Authors specified that the papillo-macular bundle could be affected earlier in the disease process also in absence of ON, and that this concurs with early Sd-OCT RNFL reduction in the thickest temporal sector in MS [40], as also seen in other neurodegenerative disorders like glaucoma [41], Parkinson’s [42] and Alzheimer’s [43] diseases.

Moreover, all sectorial WR, IR and OR MVs and MTs in MS-ON-P eyes were significantly reduced as compared to Controls. To our knowledge, no previous reports described similar investigations on Sd-OCT macular sectors. These morphological findings can suggest that in MS-ON-P eyes there is not a prevalent structural involvement of one macular sector with respect to others.

Nevertheless, this morphological impairment cannot influence the functional condition of the S, T and I sectors (that was not significantly different from Controls), as suggested by the lack of correlation between mfERG and Sd-OCT data (see Table 7). In addition, although a morphological impairment of MV and MT and a dysfunction of O-MR layers in the nasal sector were found, the absence of correlation (see Table 7) might suggest that the morphological and functional conditions are independent.

The biological mechanisms underlying the reduction of RADs in our selected group of MS-ON-P, with no previous or present signs of retinal inflammation, can only be hypothesized.

One hypothesis is that in a sub-set of MS-ON patients, a dysfunction of photoreceptors and inhibitory bipolar cells (leading to reduced mfERG RADs) is due to trans-synaptic retrograde degeneration distal to IML. Indeed, the injury that involves the IML (detectable by reduced P-ERG responses [4,5,6]) could extend more deeply, impairing outer retinal function. This hypothesis, however, on one side is not confirmed by animal studies on the retinal changes after optic nerve transection. In fact, Hollander et al. showed that only the IML are impaired at the light and electron microscopy after optic nerve damage [44]. On the other hand, a full body of evidence in humans supports the fact that trans-synaptic degeneration affects the dorsal lateral geniculate nucleus, but stops at the INL, where the bipolars reside, acting as a potential physiological protective barrier against neurodegeneration [45]. This prominent role of INL is also justified by the occurrence of dynamic and transient phenomena, also in absence of ON, as the microcystic inner retina edema often seen in MS [46,47]. At this level, the homoeostasis of the bipolar system becomes crucial for neurodegeneration processes in MS. Our evidence might suggest that when there is a poor recovery of visual acuity after an ON event, an unbalanced function of the bipolar cells system may occur and this can be detected by recording a reduction in amplitude of mfERG responses.

A second hypothesis that can explain the reduction of mfERG RADs in MS-ON-P patients is a process related to autoimmunity. For instance, in some MS patients with autoantibodies against the retinal protein α-enolase, a reduction of ERG responses has been found [48]. In addition, in validated MS mouse models of ON, it has been reported early altered synaptic vesicle cycling in ribbon synapses, located between outer and inner retinal layers, which are likely targeted by an auto-reactive immune system process [49]. Two adhesion proteins (CASPR1/CNTN1) [50], present at the level of both the paranodal region of myelinated nerves as well as at retinal ribbon synapses [49], could be the specific targets of the auto-immune response in experimental animal models.

Of course, all previous electrophysiological studies done by recording Ff-ERG or flicker ERG in MS eyes, and almost unanimously finding subnormal cone-driven bipolar cell function [16,22,51], are not comparable to our mfERG findings. This is based on the knowledge that mfERG responses are derived from cells localized into the central retina (in our study within the 25 central retinal degrees) [51], whereas Ff-ERG or flicker ERG responses are generated by the preganglionic elements of the whole retina [9].

## 5. Conclusions

In conclusion, in our study we detected an absence of mfERG abnormalities in MS patients without and with ON followed by full recovery of BCVA. Thus, our results suggest that in MS the function of preganglionic elements located in the O-MR layers is not modified by the occurrence of ON itself. By contrast, the MS neurodegenerative processes could induce a dysfunction of the preganglionic elements of the fovea and the retinal nasal sector after an event of ON followed by permanent impairment of visual acuity (poor recovery of BCVA after ON). This functional impairment was associated, but not correlated, with OR and IR structural changes. In order to better understand the role of middle retinal elements in this process, further studies on both experimental [37,48] and clinical sides [20,44] are needed.

## Figures and Tables

**Table 1:** Demographic and clinical features in Controls, Multiple Sclerosis patients without Optic Neuritis (MS-noON), with Optic Neuritis and good recovery of best corrected visual acuity (MS-ON-G) and with Optic Neuritis and poor recovery of best corrected visual acuity (MS-ON-P).

	Control (N ^a^ = 41) (Mean ± 1SD ^b^)	MS-noON (N ^a^ = 41) (Mean ± 1SD ^b^)	MS-ON-G (N ^a^ = 27) (Mean ± 1SD ^b^)	MS-ON-P (N ^a^ = 20) (Mean ± 1SD ^b^)
Age (years)	40.64 ± 4.83	41.32 ± 3.72	39.92 ± 4.86 ^§^	41.08 ± 4.66 ^§,#^
Male/Female (Ratio)	15/26 (0.57)	14/27 (0.51)	10/17 (0.58)	8/12 (0.66)
MS-DD ^c^ (years)	-	8.53 ± 4.19	9.06 ± 5.58 ^§^	9.96 ± 6.03 ^§,#^
EDSS ^d^ score	-	1.43 ± 1.06	1.53 ± 1.22 ^§^	1.49 ± 1.18 ^§,#^
Number of ON ^e^ episodes	-	-	1.00 ± 0.00	1.00 ± 0.00 ^#^
Time elapsed from ON to the mfERG ^f^ and BCVA ^g^ assessments (months)	-	-	14.12 ± 2.72	15.87 ± 3.46 ^#^

^a^ N = Number of eyes of each Group; ^b^ SD = one Standard Deviation of the mean; ^c^ MS-DD = Multiple Sclerosis Disease Duration; ^d^ EDSS = Expanded Disability Status Scale; ^e^ ON = optic neuritis; ^f^ mfERG = multifocal electroretinogram; ^g^ BCVA = best corrected visual acuity; One-way analysis of variance between Groups: ^§^ *p* > 0.01 vs. Control and MS-noON Groups, ^#^ *p* > 0.01 vs. MS-ON-G Group.

**Table 2:** Multifocal electroretinogram ring analysis in Control (C) eyes and in Multiple Sclerosis patients without Optic Neuritis (MS-noON), with optic neuritis followed by good recovery of best corrected visual acuity (BCVA) (MS-ON-G) or poor recovery of BCVA (MS-ON-P).

		Ring 1: 0–5 Degrees			Ring 2: 5–10 Degrees			Ring 3: 10–15 Degrees			Ring 4: 15–20 Degrees			Ring 5: 20–25 Degrees		
		N1 IT ^a^	P1 IT ^a^	RAD ^b^	N1 IT ^a^	P1 IT ^a^	RAD ^b^	N1 IT ^a^	P1 IT ^a^	RAD ^b^	N1 IT ^a^	P1 IT ^a^	RAD ^b^	N1 IT ^a^	P1 IT ^a^	RAD ^b^
**Controls** **N ^d^ = 41**	Mean	14.693	29.785	56.137	13.863	28.793	22.037	12.890	28.110	12.012	12.815	28.168	8.724	13.459	28.944	7.129
	SD ^c^	2.666	2.678	10.771	1.712	1.349	4.816	1.312	1.394	3.090	2.396	1.331	2.090	1.247	1.611	1.778
**MS-noON** **N ^d^ = 41**	Mean	15.078	30.035	54.273	13.743	28.393	21.505	13.193	27.413	12.525	13.115	28.455	9.050	13.198	28.650	7.535
	SD ^c^	2.383	1.958	11.665	2.177	1.661	4.556	2.505	3.076	3.409	1.020	1.285	2.573	1.156	1.115	2.321
**A ^e^ vs. C**	f(1.81)	0.491	0.234	0.568	0.078	1.432	0.262	0.464	0.913	0.063	0.582	1.002	0.409	0.949	0.902	0.812
	P	0.487	0.638	0.453	0.782	0.235	0.610	0.498	0.343	0.810	0.449	0.319	0.525	0.332	0.345	0.372
**MS-ON-G** **N ^d^ = 27**	Mean	15.748	30.156	53.467	13.741	29.104	21.081	12.800	28.041	12.344	13.089	28.585	8.926	12.963	28.596	7.511
	SD ^c^	2.934	2.739	11.053	1.903	1.761	5.121	1.522	1.831	2.755	1.042	1.484	1.906	0.692	1.196	1.622
**A ^e^ vs. C**	f(1.67)	2.371	0.029	1.000	0.071	0.672	0.609	0.068	0.032	0.262	0.332	1.423	0.183	3.591	0.879	0.799
	P	0.128	0.563	0.321	0.787	0.415	0.463	0.796	0.859	0.611	0.570	0.238	0.676	0.063	0.352	0.376
**A ^e^ vs. MS-noON**	f(1.67)	1.072	0.042	0.079	0.000	2.841	0.164	0.532	0.913	0.063	0.013	0.153	0.042	0.932	0.029	0.009
	P	0.304	0.834	0.776	1.000	0.097	0.693	0.470	0.343	0.810	0.907	0.703	0.836	0.337	0.861	0.954
**MS-ON-P** **N ^d^ = 20**	Mean	15.657	30.012	43.136	13.976	29.464	21.362	12.984	28.524	13.486	13.002	28.648	8.322	13.892	28.027	6.994
	SD ^c^	2.572	2.923	10.964	2.023	1.941	6.013	1.937	1.641	3.904	2.474	1.823	3.566	1.721	2.526	2.843
**A ^e^ vs. C**	f(1.60)	1.802	0.091	19.36	0.053	2.473	0.221	0.053	1.053	2.570	0.084	1.363	0.311	1.262	2.962	0.051
	P	0.185	0.764	0.000 ^f^	0.821	0.121	0.638	0.824	0.309	0.115	0.778	0.248	0.581	0.267	0.090	0.821
**A ^e^ vs. MS-noON**	f(1.60)	0.753	0.002	12.73	0.162	5.003	0.012	0.111	2.283	0.972	0.064	0.233	0.831	3.482	1.812	0.630
	P	0.389	0.971	0.000 ^f^	0.690	0.029	0.918	0.744	0.137	0.328	0.801	0.634	0.366	0.067	0.184	0.431
**A ^e^ vs. MS-ON-G**	f(1.46)	0.012	0.033	11.41	0.172	0.443	0.033	0.133	0.871	1.383	0.033	0.021	0.562	6.552	1.062	0.626
	P	0.912	0.862	0.002 ^f^	0.686	0.510	0.864	0.717	0.355	0.245	0.870	0.897	0.458	0.014	0.309	0.434

^a^ IT = Implicit Time (measured in msec); ^b^ RAD = N1-P1 Response Amplitude Density (measured in ηV/degree^2^); ^c^ SD = one Standard Deviation of the mean; ^d^ N = Number of eyes of each Group; ^e^ A = one-way analysis of variance. ^f^ *p* Values < 0.01 were considered as statistically significant for Group comparisons.

**Table 3:** Multifocal electroretinogram sector analysis within the 0–15 central degrees in Control (C) eyes and in Multiple Sclerosis patients without Optic Neuritis (MS-noON), with optic neuritis followed by good recovery of best corrected visual acuity (BCVA) (MS-ON-G), or poor recovery of BCVA (MS-ON-P).

		0–15 Central Degrees Superior Sector			0–15 Central Degrees Temporal Sector			0–15 Central Degrees Inferior Sector			0–15 Central Degrees Nasal Sector		
		N1 IT ^a^	P1 IT ^a^	RAD ^b^	N1 IT ^a^	P1 IT ^a^	RAD ^b^	N1 IT ^a^	P1 IT ^a^	RAD ^b^	N1 IT ^a^	P1 IT ^a^	RAD ^b^
**Controls** **N ^d^ = 41**	Mean	13.266	28.800	17.910	13.251	28.917	17.944	13.373	28.327	17.573	13.195	27.698	19.039
	SD ^c^	1.712	1.588	4.254	1.659	1.776	4.746	1.747	1.432	4.424	1.742	1.300	4.406
**MS-noON** **N ^d^ = 41**	Mean	13.758	28.319	17.442	13.972	28.508	18.119	13.881	27.997	16.489	13.831	27.506	19.369
	SD ^c^	2.058	1.438	3.982	1.996	1.677	4.094	2.167	1.525	4.131	1.679	1.587	4.508
**A ^e^ vs. C**	f(1.81)	1.379	2.069	0.262	3.158	1.148	0.029	1.368	1.018	1.308	2.028	0.362	0.108
	P	0.243	0.154	0.608	0.079	0.287	0.859	0.246	0.316	0.255	0.096	0.551	0.738
**MS-ON-G** **N ^d^ = 27**	Mean	13.256	28.459	17.241	13.774	28.356	18.278	13.419	28.648	17.007	13.570	28.037	17.933
	SD ^c^	1.430	1.554	3.932	2.042	2.237	4.843	2.027	1.289	4.129	1.610	1.806	4.246
**A ^e^ vs. C**	f(1.67)	0.003	0.758	0.432	1.352	1.320	0.079	0.009	0.878	0.282	0.801	0.809	1.002
	P	0.988	0.385	0.516	0.250	0.255	0.779	0.921	0.351	0.598	0.374	0.371	0.321
**A ^e^ vs. MS-noON**	f(1.67)	1.222	0.138	0.039	0.162	0.102	0.019	0.779	3.340	0.258	0.408	1.632	1.642
	P	0.274	0.705	0.838	0.693	0.750	0.885	0.381	0.072	0.615	0.526	0.206	0.205
**MS-ON-P** **N ^d^ = 20**	Mean	13.519	29.004	17.828	13.987	28.763	17.874	13.287	28.736	17.232	14.122	28.006	14.892
	SD ^c^	2.391	1.738	5.008	2.674	2.222	4.586	2.562	2.876	5.023	1.936	1.964	3.225
**A ^e^ vs. C**	f(1.60)	0.222	0.212	0.002	1.75	0.092	0.002	0.022	0.552	0.072	3.542	0.532	14.00
	P	0.637	0.645	0.947	0.191	0.771	0.957	0.878	0.459	0.788	0.065	0.468	0.000 ^f^
**A ^e^ vs. MS-noON**	f(1.60)	0.161	2.663	0.111	0.002	0.253	0.042	0.901	1.731	0.383	0.372	1.642	15.73
	P	0.688	0.108	0.745	0.980	0.619	0.834	0.348	0.193	0.542	0.548	0.205	0.000 ^f^
**A ^e^ vs. MS-ON-G**	f(1.46)	0.222	1.284	0.202	0.101	0.386	0.081	0.042	0.022	0.032	1.142	0.002	7.192
	P	0.641	0.264	0.655	0.758	0.539	0.774	0.345	0.888	0.867	0.292	0.956	0.009 ^f^

^a^ IT = Implicit Time (measured in msec); ^b^ RAD = N1-P1 Response Amplitude Density (measured in ηV/degree^2^); ^c^ SD = one Standard Deviation of the mean; ^d^ N = Number of eyes of each Group; ^e^ A = one-way analysis of variance. ^f^ *p* Values < 0.01 were considered as statistically significant for Group comparisons.

**Table 4:** Multifocal electroretinogram sector analysis within the 0–25 central degrees in Control (C) eyes and in Multiple Sclerosis patients without Optic Neuritis (MS-noON), with optic neuritis followed by good recovery of best corrected visual acuity (BCVA) (MS-ON-G), or poor recovery of BCVA (MS-ON-P).

		0–25 Degrees Superior Sector			0–25 Degrees Temporal Sector			0–25 Degrees Inferior Sector			0–25 Degrees Nasal Sector		
		N1 IT ^a^	P1 IT ^a^	RAD ^b^	N1 IT ^a^	P1 IT ^a^	RAD ^b^	N1 IT ^a^	P1 IT ^a^	RAD ^b^	N1 IT ^a^	P1 IT ^a^	RAD ^b^
**Controls (N ^d^ = 41)**	Mean	13.090	28.783	9.759	13.402	28.027	9.176	13.283	28.680	8.132	13.268	27.985	9.388
	SD ^c^	1.504	1.357	2.463	1.240	1.405	2.793	1.378	1.613	2.217	1.479	1.227	2.197
**MS-noON (N ^d^ = 41)**	Mean	13.133	28.258	10.192	13.336	28.467	9.181	13.394	28.281	7.994	12.964	27.661	9.994
	SD ^c^	1.154	1.240	2.789	1.365	1.132	2.606	1.793	1.640	2.558	0.858	1.217	3.022
**A ^e^ vs. C**	f(1.81)	0.021	3.339	0.561	0.049	2.439	0.001	0.100	1.229	0.069	1.129	1.442	1.082
	P	0.896	0.071	0.450	0.819	0.122	0.993	0.754	0.270	0.795	0.258	0.234	0.302
**MS-ON-G (N ^d^ = 27)**	Mean	12.904	28.763	10.022	13.333	28.307	9.437	13.296	28.900	8.322	12.793	27.856	9.552
	SD ^c^	1.121	1.055	1.857	1.775	1.407	2.224	1.308	1.450	2.246	0.998	1.260	1.963
**A ^e^ vs. C**	f(1.67)	0.301	0.009	0.219	0.039	0.649	0.168	0.002	0.329	0.118	2.761	0.182	0.104
	P	0.585	0.949	0.638	0.851	0.424	0.685	0.969	0.569	0.732	0.101	0.676	0.755
**A ^e^ vs. MS-noON**	f(1.67)	0.659	3.028	0.079	0.022	0.272	0.178	0.059	2.538	0.092	0.567	0.409	0.448
	P	0.421	0.086	0.782	0.994	0.607	0.676	0.808	0.116	0.589	0.454	0.526	0.504
**MS-ON-P N ^d^ = 20**	Mean	13.834	28.916	9.786	14.003	28.237	9.924	13.977	28.471	8.976	13.219	28.104	9.812
	SD ^c^	1.345	1.723	2.923	2.656	1.579	3.512	1.422	1.765	2.784	1.806	3.245	2.782
**A ^e^ vs. C**	f(1.60)	3.522	0.11	0.00	1.437	0.28	0.812	3.342	0.211	1.644	0.012	0.040	0.422
	P	0.066	0.744	0.970	0.231	0.601	0.371	0.073	0.647	0.205	0.911	0.838	0.520
**A ^e^ vs. MS-noON**	f(1.60)	4.454	2.910	0.281	1.690	0.430	0.871	1.615	0.173	1.874	0.564	0.60	0.051
	P	0.039	0.093	0.601	0.198	0.517	0.356	0.209	0.680	0.177	0.456	0.442	0.822
**A ^e^ vs. MS-ON-G**	f(1.46)	6.673	0.143	0.111	1.073	0.031	0.345	2.892	0.845	0.082	1.076	0.132	0.141
	P	0.013	0.708	0.737	0.305	0.874	0.564	0.096	0.365	0.377	0.307	0.718	0.709

^a^ IT = Implicit Time (measured in msec); ^b^ RAD = N1-P1 Response Amplitude Density (measured in ηV/degree^2^); ^c^ SD = one Standard Deviation of the mean; ^d^ N = Number of eyes of each Group; ^e^ A = one-way analysis of variance.

**Table 5:** Spectral domain-Optical Coherence Tomography macular volume (MV) (A) and macular thickness (MT) (B) segmentation analysis in Control (C) eyes and in Multiple Sclerosis patients with Optic Neuritis and reduced recovery of best corrected visual acuity (MS-ON-P).

**A**		**WR-MV (mm^3^)**				**IR-MV (mm^3^)**				**OR-MV (mm^3^)**			
		**AREA 1**	**AREA 2**	**AREA 3**	**AREA 1 + 2 + 3**	**AREA 1**	**AREA 2**	**AREA 3**	**AREA 1 + 2 + 3**	**AREA 1**	**AREA 2**	**AREA 3**	**AREA 1 + 2 + 3**
**Controls** **N ^b^ = 41**	Mean	0.212	2.025	3.656	5.893	0.065	0.824	1.451	2.342	0.146	1.201	2.205	3.552
	SD ^a^	0.019	0.135	0.245	0.372	0.018	0.075	0.127	0.199	0.016	0.088	0.233	0.328
**MS-ON-P** **N ^b^ = 20**	Mean	0.189	1.599	2.087	3.875	0.056	0.632	1.217	1.905	0.133	0.967	2.008	3.108
	SD ^a^	0.015	0.086	1.412	0.289	0.012	0.038	0.122	0.272	0.008	0.174	0.077	0.131
**A ^c^ vs. C**	f(1.60)	22.422	165.532	40.471	453.482	0.112	115.824	46.800	50.661	11.701	49.074	13.482	33.771
	P	0.000 ^d^	0.000 ^d^	0.000 ^d^	0.000 ^d^	0.742	0.000 ^d^	0.000 ^d^	0.000 ^d^	0.001 ^d^	0.000 ^d^	<0.01 ^d^	0.000 ^d^
**B**		**WR-MT (μ)**				**IR-MT (μ)**				**OR-MT (μ)**			
		**AREA 1**	**AREA 2**	**AREA 3**		**AREA 1**	**AREA 2**	**AREA 3**		**AREA 1**	**AREA 2**	**AREA 3**	
**Controls** **N ^b^ = 41**	Mean	263.866	327.674	299.975		81.134	138.427	114.422		182.732	189.247	185.553	
	SD ^a^	12.512	12.913	9.572		10.561	8.946	6.566		9.884	9.983	7.002	
**MS-ON-P** **N ^b^ = 20**	Mean	243.834	275.179	263.253		69.417	106.667	96.833		174.417	168.512	166.417	
	SD ^a^	12.999	13.665	10.146		10.227	7.183	9.737		7.225	7.816	5.979	
**A ^c^ vs. C**	f(1.60)	38.672	213.901	190.280		16.893	191.321	69.202		11.199	86.811	110.000	
	P	0.000 ^d^	0.000 ^d^	0.000 ^d^		0.000 ^d^	0.000 ^d^	0.000 ^d^		0.001 ^d^	0.000 ^d^	0.000 ^d^	

WR-MV = Whole Retinal Macular Volume; IR-MV = Inner Retinal Macular Volume; OR-MV = Outer Retinal Macular Volume; WR-MT = Whole Retinal Macular Thickness; IR-MT = Inner Retinal Macular Thickness; OR-MT = Outer Retinal Macular Thickness; μ = micron; Area 1 = 1 mm centered to the fovea; Area 2 = annular area 1–3 mm centered to the fovea; Area 3 = annular area 3–6 mm centered to the fovea; Area 1 + 2 + 3 = whole area within 6 mm; ^a^ SD = one Standard Deviation of the mean; ^b^ N = Number of eyes of each Group; ^c^ A = one-way analysis of variance. ^d^ *p* Values < 0.01 were considered as statistically significant for Group comparisons.

**Table 6:** Spectral domain-Optical Coherence Tomography macular volume (MV) (A) and macular thickness (MT) (B) sectorial segmentation analysis in Control (C) eyes and in Multiple Sclerosis patients with Optic Neuritis and reduced recovery of best corrected visual acuity (MS-ON-P).

		**SUPERIOR SECTOR**			**TEMPORAL SECTOR**			**INFERIOR SECTOR**			**NASAL SECTOR**		
**A**		**WR-MV**	**IR-MV**	**OR-MV**	**WR-MV**	**IR-MV**	**OR-MV**	**WR-MV**	**OR-MV**	**IR-MV**	**WR-MV**	**IR-MV**	**OR-MV**
		**(mm^3^)**	**(mm^3^)**	**(mm^3^)**	**(mm^3^)**	**(mm^3^)**	**(mm^3^)**	**(mm^3^)**	**(mm^3^)**	**(mm^3^)**	**(mm^3^)**	**(mm^3^)**	**(mm^3^)**
**Controls** **N ^b^ = 41**	Mean	0.551	0.214	0.337	0.537	0.203	0.334	0.551	0.216	0.335	0.579	0.231	0.348
	SD ^a^	0.029	0.017	0.019	0.031	0.015	0.02	0.028	0.012	0.021	0.048	0.018	0.024
**MS-ON-P** **N ^b^ = 20**	Mean	0.475	0.168	0.307	0.479	0.172	0.307	0.487	0.177	0.310	0.478	0.175	0.303
	SD ^a^	0.029	0.021	0.011	0.028	0.012	0.014	0.021	0.016	0.016	0.028	0.015	0.015
**A ^c^ vs. C**	f(1.60)	92.321	84.173	42.642	50.022	64.942	29.312	81.756	113.55	22.032	75.577	144.312	58.801
	P	0.000 ^d^	0.000 ^d^	0.000 ^d^	0.000 ^d^	0.000 ^d^	0.000 ^d^	0.000 ^d^	0.000 ^d^	0.000 ^d^	0.000 ^d^	0.000 ^d^	0.000 ^d^
**B**		**WR-MT**	**IR-MT**	**OR-MT**	**WR-MT**	**IR-MV**	**OR-MT**	**WR-MT**	**OR-MT**	**IR-MT**	**WR-MT**	**IR-MT**	**OR-MT**
		**(μ)**	**(μ)**	**(μ)**	**(μ)**	**(μ)**	**(μ)**	**(μ)**	**(μ)**	**(μ)**	**(μ)**	**(μ)**	**(μ)**
**Controls** **N ^b^ = 41**	Mean	297.124	111.358	185.766	292.804	108.243	184.561	297.078	112.518	184.56	306.789	117.604	189.185
	SD ^a^	8.573	7.217	6.755	8.239	7.862	6.561	6.072	6.756	6.366	7.541	8.822	6.341
**MS-ON-P** **N ^b^ = 20**	Mean	271.297	94.442	176.857	271.135	92.052	178.995	268.411	94.191	174.221	274.021	97.834	176.190
	SD ^a^	6.580	7.065	5.417	7.742	6.325	5.444	8.341	9.344	7.085	8.033	7.898	5.253
**A ^c^ vs. C**	f(1.60)	140.612	68.222	26.427	96.632	64.326	10.755	233.066	76.457	32.933	243.281	72.150	69.390
	P	0.000 ^d^	0.000 ^d^	0.000 ^d^	0.000 ^d^	0.000 ^d^	0.002 ^d^	0.000 ^d^	0.000 ^d^	0.000 ^d^	0.000 ^d^	0.000 ^d^	0.000 ^d^

WR-MV = Whole Retinal Macular Volume; IR-MV = Inner Retinal Macular Volume; OR-MV = Outer Retinal Macular Volume; WR-MT = Whole Retinal Macular Thickness; IR-MT = Inner Retinal Macular Thickness; OR-MT = Outer Retinal Macular Thickness; μ = micron; ^a^ SD = one Standard Deviation of the mean; ^b^ N = Number of eyes of each Group; ^c^ A = one-way analysis of variance; ^d^ *p* Values < 0.01 were considered as statistically significant for Group comparisons.

**Table 7:** Linear correlation (Pearson’s Test) between multifocal electroretinogram values from ring analysis (A) and from sector analysis 1 (0–15 central degrees) (B) and the corresponding Spectral domain-Optical Coherence Tomography macular volume (MV) and macular thickness (MT) segmentation analysis individual values in Multiple Sclerosis patients with Optic Neuritis and reduced recovery of best corrected visual acuity (MS-ON-P).

**A**	**AREA 1 vs. mfERG Ring1**			**AREA 2 vs. mfERG Ring 2**			**AREA 3 vs. mfERG Ring 3**			**AREA 1 + 2 + 3 vs. Rings 1 + 2 + 3**		
	**N1 IT ^a^**	**P1 IT ^a^**	**RAD ^b^**	**N1 IT ^a^**	**P1 IT ^a^**	**RAD ^b^**	**N1 IT ^a^**	**P1 IT ^a^**	**RAD ^b^**	**N1 IT ^a^**	**P1 IT ^a^**	**RAD ^b^**
	**r; p ^c^**	**r; p ^c^**	**r; p ^c^**	**r; p ^c^**	**r; p ^c^**	**r; p ^c^**	**r; p ^c^**	**r; p ^c^**	**r; p ^c^**	**r; p ^c^**	**r; p ^c^**	**r; p ^c^**
**WR-MV**	0.198; 0.535	−0.055; 0.862	0.512; 0.088	0.192; 0.549	−0.103; 0.748	0.245; 0.441	−0.324; 0.303	−0.509; 0.090	−0.163; 0.611	−0.169; 0.599	−0.474; 0.118	0.037; 0.907
**IR-MV**	0.338; 0.282	0.086; 0.794	0.511; 0.090	0.132; 0.683	−0.160; 0.621	0.283; 0.372	−0.288; 0.365	−0.437; 0.156	−0.061; 0.851	0.185; 0.565	−0.265; 0.405	0.261; 0.413
**OR-MV**	0.037; 0.910	−0.193; 0.548	0.443; 0.168	0.228; 0.475	−0.348; 0.267	−0.498; 0.099	−0.345; 0.271	−0.567; 0.055	−0.309; 0.328	−0.173; 0.591	−0.308; 0.331	−0.074; 0.819
**WR-MT**	0.201; 0.532	−0.053; 0.870	0.409; 0.131	0.184; 0.567	−0.100; 0.756	0.254; 0.426	−0.324; 0.304	−0.517; 0.086	−0.167; 0.604	-	-	-
**IR-MT**	0.331; 0.293	0.073; 0.822	0.507; 0.093	0.134; 0.677	−0.176; 0.585	0.281; 0.376	−0.298; 0.347	−0.435; 0.158	−0.061; 0.851	-	-	-
**OR-MT**	0.042; 0.896	−0.181; 0.574	0.369; 0.204	0.223; 0.486	−0.021; 0.947	0.169; 0.600	−0.336; 0.285	−0.571; 0.052	−0.309; 0.329	-	-	-
**B**	**SUPERIOR SECTOR**			**TEMPORAL SECTOR**			**INFERIOR SECTOR**			**NASAL SECTOR**		
	**N1 IT ^a^**	**P1 IT ^a^**	**RAD ^b^**	**N1 IT ^a^**	**P1 IT ^a^**	**RAD ^b^**	**N1 IT ^a^**	**P1 IT ^a^**	**RAD ^b^**	**N1 IT ^a^**	**P1 IT ^a^**	**RAD ^b^**
	**r; p ^c^**	**r; p ^c^**	**r; p ^c^**	**r; p ^c^**	**r; p ^c^**	**r; p ^c^**	**r; p ^c^**	**r; p ^c^**	**r; p ^c^**	**r; p ^c^**	**r; p ^c^**	**r; p ^c^**
**WR-MV**	0.167; 0.602	−0.113; 0.726	0.281; 0.376	−0.331; 0.292	−0.119; 0.711	−0.331; 0.292	−0.045; 0.888	−0.569; 0.053	0.193; 0.547	−0.119; 0.710	−0.036; 0.910	−0.056; 0.861
**IR-MV**	0.156; 0.627	−0.166; 0.606	0.349; 0.266	−0.363; 0.245	−0.078; 0.809	−0.363; 0.245	0.100; 0.756	−0.341; 0.277	0.171; 0.595	−0.169; 0.598	−0.109; 0.734	0.082; 0.798
**OR-MV**	0.163; 0.611	0.020; 0.949	0.085; 0.791	−0.125; 0.698	−0.063; 0.845	−0.125; 0.698	−0.168; 0.601	−0.228; 0.407	0.073; 0.819	−0.034; 0.916	−0.060; 0.852	−0.224; 0.483
**WR-MT**	−0.162; 0.614	0.001; 0.999	0.742; 0.006	−0.159; 0.620	−0.418; 0.175	−0.159; 0.620	0.110; 0.731	−0.549; 0.064	0.131; 0.684	−0.099; 0.759	−0.137; 0.669	−0.074; 0.817
**IR-MT**	0.145; 0.650	−0.208; 0.516	0.313; 0.321	−0.374; 0.230	−0.082; 0.799	−0.374; 0.230	0.265; 0.404	−0.318; 0.312	0.201; 0.529	−0.117; 0.716	−0.093; 0.771	0.052; 0.870
**OR-MT**	0.060; 0.852	0.002; 0.994	0.088; 0.785	−0.325; 0.302	−0.164; 0.608	−0.325; 0.302	−0.028; 0.929	−0.391; 0.312	0.055; 0.865	−0.065; 0.840	−0.159; 0.621	−0.186; 0.561

WR-MV = Whole Retinal Macular Volume; IR-MV = Inner Retinal Macular Volume; OR-MV = Outer Retinal Macular Volume; WR-MT = Whole Retinal Macular Thickness; IR-MT = Inner Retinal Macular Thickness; OR-MT = Outer Retinal Macular Thickness; μ = micron; Area 1 = 1 mm centered to the fovea; Area 2 = annular area 1–3 mm centered to the fovea; Area 3 = annular area 3–6 mm centered to the fovea; Area 1 + 2 + 3 = whole area within 6 mm. ^a^ IT = Implicit Time (measured in msec); ^b^ RAD = N1-P1 Response Amplitude Density (measured in ηV/degree^2^); ^c^ *p* values < 0.01 were considered as statistically significant.

## Abbreviations

MS	multiple sclerosis
ON	optic neuritis
MS-noON	multiple sclerosis patients without optic neuritis
MS-ON-G	multiple sclerosis patients with optic neuritis followed by good recovery of best corrected visual acuity
MS-ON-P	multiple sclerosis patients with optic neuritis followed by poor recovery of best corrected visual acuity
BCVA	best corrected visual acuity
MfERG	multifocal electroretinogram
IT	implicit time
RAD	response amplitude density
P-ERG	pattern electroretinogram
Ff-ERG	Full-field electroretinogram
F-ERG	focal electroretinogram
IML	innermost retinal layers
O-MR	outer and in middle retinal
SD	one standard deviation of the mean
N	number of eyes of each group
A	one-way analysis of variance
MV	macular volume
MT	macular thickness
WR	whole retina
IR	inner retina
OR	outer retina
S-S	sector-superior
S-T	sector-temporal
S-I	sector-inferior
S-N	sector -nasal
